# Perspective on human pluripotent stem cell‐derived cardiomyocytes in heart disease modeling and repair

**DOI:** 10.1002/sctm.19-0340

**Published:** 2020-07-29

**Authors:** Qiang Li, Jia Wang, Qiang Wu, Nan Cao, Huang‐Tian Yang

**Affiliations:** ^1^ CAS Key Laboratory of Tissue Microenvironment and Tumor, Laboratory of Molecular Cardiology Shanghai Institute of Nutrition and Health, University of Chinese Academy of Sciences (CAS) Shanghai People's Republic of China; ^2^ Institute for Stem Cell and Regeneration, CAS Beijing People's Republic of China; ^3^ Program of Stem Cells and Regenerative Medicine, The Fifth Affiliated Hospital, Zhongshan School of Medicine, Sun Yat‐Sen University Guangdong People's Republic of China; ^4^ Key Laboratory for Stem Cells and Tissue Engineering (Sun Yat‐Sen University), Ministry of Education Guangzhou People's Republic of China

**Keywords:** cardiac, cell transplantation, differentiation, embryonic stem cells (ESCs), induced pluripotent stem cells (iPSCs), pluripotent stem cells, tissue regeneration

## Abstract

Heart diseases (HDs) are the leading cause of morbidity and mortality worldwide. Despite remarkable clinical progress made, current therapies cannot restore the lost myocardium, and the correlation of genotype to phenotype of many HDs is poorly modeled. In the past two decades, with the rapid developments of human pluripotent stem cell (hPSC) biology and technology that allow the efficient preparation of cardiomyocytes from individual patients, tremendous efforts have been made for using hPSC‐derived cardiomyocytes in preclinical and clinical cardiac therapy as well as in dissection of HD mechanisms to develop new methods for disease prediction and treatment. However, their applications have been hampered by several obstacles. Here, we discuss recent advances, remaining challenges, and the potential solutions to advance this field.

1

**Significance statement**

With the ability to generate highly enriched populations of multiple cardiac cell types from human pluripotent stem cells, it has never been closer for researchers in this field to test their applications in repairing damaged myocardium, modeling human congenital heart diseases, and screening for therapeutic chemicals. This perspective outlines the successful experiences and limitations of current studies and summarizes some application barriers as well as potential directions to enable this field to move forward.

## 2 INTRODUCTION

Heart diseases (HDs) remain the most common cause of morbidity and mortality worldwide.^1^, ^2^ HDs encompass a broad range of disorders extending from myocardial infarction (MI) to heritable cardiomyopathies and heart failure (HF), either caused by pathological insults or inherited DNA sequence variants.^3^, ^4^, ^5^ Rapid advances in the stem cell field have lightened the hope of regenerating the heart by transplantation of stem cell‐derived cardiomyocytes (CMs).^1^, ^2^, ^6^, ^7^ To date, the only robust source for efficient generation of authentic human cardiomyocytes is human pluripotent stem cells (hPSCs), including embryonic stem cells (hESCs), and induced pluripotent stem cells (hiPSCs).^8^, ^9^, ^10^ Cardiovascular progenitor cells (CVPCs), endothelial cells (ECs), smooth muscle cells (SMCs), epicardial cells, cardiac fibroblasts (CFs), and mesenchymal stem cells (MSCs) are also successfully generated from hPSCs.^11^, ^12^, ^13^, ^14^, ^15^, ^16^, ^17^ Upon these progresses, hPSC‐derived cardiac lineage cells have been studied as candidates for cell therapy in small and large animal ischemic heart disease models and severe ischemic HF patients.^1^, ^2^, ^6^, ^18^, ^19^, ^20^, ^21^, ^22^, ^23^, ^24^, ^25^, ^26^ Although the outcomes are encouraging, several obstacles need to be overcome before moving toward clinical application. In addition, advancements in hPSC‐based disease modeling have not only shed new lights on the genetic and/or molecular basis of many cardiac diseases but also established cellular phenotypes characterizing HDs, revealing tremendous potential in the phenotypic approach of drug discovery.^6^, ^27^ Here, we overview recent advances, challenges, and potential solutions in the application of hPSC‐derived cardiomyocytes in these fields, with the focus on cardiac therapy, heart disease modeling, and drug discovery.

## 3 hPSC‐DERIVED CARDIOMYOCYTES FOR INFARCT REPAIR

### 3.1 Preclinical and clinical studies

Owing to the restricted regenerative capacity of adult hearts, the massive loss of cardiac tissue, particularly of CMs, following MI is replaced by fibrotic tissues, leading to adverse remolding and fatal HF.^1^, ^6^ hPSC‐derived cardiomyocytes have emerged as candidates for cardiac regenerative therapy.^6^ Intra‐myocardial injection of hESC‐CMs at 7 to 10 days after either ischemia/reperfusion (I/R) or permanent MI improves cardiac remodeling and functional performance in rat hearts.^18^, ^19^, ^20^ Similar functional efficacy of transplanted hiPSC‐CMs has been observed in the rodent infarcted model.^28^ Transplanted hESC‐CMs also electrically couple and suppress arrhythmias in injured guinea‐pig hearts.^29^ Furthermore, hPSC‐CVPCs^12^, ^13^, ^30^ benefit heart function when delivered either at acute^23^ or subacute^31^ phase of MI in rodent animals.

To confirm the feasibility and efficacy of these repairing strategies, recent studies have shifted toward the large animal models.^1^, ^6^, ^22^, ^32^ In adult pigtail macaques, intramyocardial injection of 0.75 billion to 1 billion of hESC‐CMs at 2 weeks after myocardial I/R showed remuscularized CM grafts, improved blood supply, electromechanical integration, and supported failing hearts but without teratoma formation over a 3‐month period.^21^, ^22^ We also found that intramyocardial injection of 10 million hESC‐CVPCs into acutely infarcted cynomolgus monkey heart improved the cardiac function.^25^ In addition, the first clinical trial of hESC‐CVPC therapy in severe ischemic HF patients during a coronary artery bypass procedure did not detect tumor and arrhythmias with a median follow‐up of 18 months.^2^, ^24^ The findings support the hope that these cells might be effective candidates for myocardial repair, whereas substantial studies are required to determine the feasibility and safety of these cells.

### 3.2 Mechanisms of hPSC‐derived cardiomyocytes for infarct repair

The initial purpose of hPSC‐CM therapy is to directly replace MI‐induced lost tissue. This is encouraged by the observations that the transplanted hPSC‐CMs or hPSC‐CVPCs to the rodent infarcted hearts formed stable CM grafts^18^, ^33^, ^34^ or differentiated into cardiomyocytes in the myocardial scar.^31^ Substantial amounts of remuscularized hESC‐CMs are detected in a macaque model of heart failure, and the grafts form electromechanical junctions with the host hearts.^21^, ^22^ However, our study has shown that transplanted hESC‐CVPCs at 30 minutes post‐MI were not detected 20 weeks later in the nonhuman primate hearts,^25^ suggesting a critical role of paracrine mechanisms in promoting cardiac repair.^23^ Many factors, including the different disease models, cell dose and types, timing of cell delivery, and immunosuppressive agents, might contribute to the discrepancy in the graft survival. In most studies, the limited engraftment of transplanted cells could not explain the obvious benefits of cardiac repair. The functional beneficial effects of hPSC‐CVPCs or hPSC‐CMs seem to critically correlate with the improved cardiomyocyte survival, increased neovascularization, and reduced scar size.^1^, ^23^, ^25^, ^30^ These beneficial effects are attributed to cell secretions, including growth factors, cytokines,^23^ microRNAs,^35^ noncoding RNA,^36^ and extracellular vehicles (EVs)/exosomes.^36^, ^37^, ^38^ The findings suggest two major mechanisms for promoting cardiac repair, that is, the replacement of the lost myocardium, and/or stimulation of endogenous repair mechanisms by paracrine effects. Therefore, in the former case, the cells need to be grafted in large amounts and functionally couple with the host myocardium permanently, whereas in the latter case, the transient engraftment of transplanted cells is acceptable^2^ and can be partially replaced by cell‐free therapy. As various factors contribute to the infarct healing via trigger endogenous cardiac repair mechanisms, it is important to thoroughly dissect the secretome of hPSC‐cardiomyocytes and elucidate how the factors and EVs/exosomes protect the heart via interacting with endogenous cells, in order to develop new therapeutic approaches for ischemic heart disease.

### 3.3 Challenges and solutions for application of hPSC‐cardio‐myocytes in carcdiac therapy

#### 3.3.1 *Cell quality*

Cell quality including genomic stability is affected by cell lines, differentiation protocols, expansion periods, developmental stage, subpopulations, storage, and dosage, etc. It is at least in part responsible for the mixed outcomes across hPSC‐based therapy, tumorigenicity, and secretomes of transplanted cells.^6^, ^14^ For example, microRNAs in hESC‐CM/hiPSC‐CM‐derived exosomes are differentially expressed in the hypoxic condition.^36^ Therefore, systematic efforts are required to strictly control each step of cell generation, including hiPSC generation, cell line selection combining with genomic stability examination, developing small molecules to selectively eliminate hPSCs, isolating subpopulations of cardiomyocytes subpopulations that are high‐yield, clinical‐grade, and cost‐effective, and setting up criteria for each step from prior to and clinical application of hPSC‐cardiomyocytes.^6^ In addition, new approaches are required to reduce the cell heterogeneity in quality, subtype, and maturation level. For example, to improve the quality of hPSC‐CMs, Kannappan et al^39^ isolated functionally competent DNA damage‐free (DdF) cells from a population of heterogeneous hPSCs by chemically activating p53. The CMs derived from DdF cells showed superior performance over the conventional hPSC‐CMs after transplantation. The mature state of hPSC‐CMs is discussed in the “Arrhythmogenicity” section.

#### 3.3.2 *Immature cell phenotype*

Human pluripotent stem cell‐derived cardiomyocytes retain a fetal phenotype in structure and function, including small cell size, disorganized sarcomere structures, underdeveloped calcium handling, as well as weak excitability, contractility, and adrenergic sensitivity.^40^, ^41^, ^42^ In addition, the hPSC‐CMs are heterogeneous and display a certain level of experimental variability in maturation.^43^ The maturity level of hPSC‐CMs would affect engraftment, proliferation, and therapeutic efficacy in the host hearts.^44^, ^45^ In addition, the immature hPSC‐CMs express the inward channel (If), which may cause arrhythmias when implanted into the adult heart.^46^ Therefore, many efforts have been made to advance the maturation of hiPSC‐CMs, such as extracellular matrix engineering, cell alignment techniques, electrical stimulation, mechanical stretching, mitochondrial engineering, as well as microRNA and hormonal interventions.^41^, ^42^, ^47^ These approaches significantly improve the maturation of hPSC‐CMs, although not achieving the maturity seen in the adult human myocardium. Moreover, immunophenotyping is one validated approach to overcome cell heterogeneity and experimental variability. We newly identified CD36 as a cell surface marker of maturation in hPSC‐CMs, which can be used to reduce the heterogeneity and experimental variability of hPSC‐CMs to facilitate their application.^43^ To facilitate the widespread adoption, maturation‐enhancing approaches that are more scalable, cost‐effective, and capable of independent replication across laboratories need to be developed, such as using small molecules to regulate key steps of mitochondrial biogenesis and metabolism at the critical time window.^44^

It has been shown that the hPSC‐CMs at differentiation day 20 engrafted better than their day 8 or 30 counterparts.^45^ Moreover, hPSC‐CMs mature extensively when transplanted into the normal or injured adult heart.^22^, ^48^ Therefore, effective cardiac cell therapy will require careful tailoring of the optimal mature state of hPSC‐CMs to balance cell survival and the proarrhythmic risk post‐transplantation. Alternatively, combining hPSC‐CMs with biomaterials to form a 3‐dimensionally engineered heart tissue (EHT) enhances the viability, functional maturation, and electromechanical coupling of hPSC‐CMs. This may lead us into a new era of cardiac cell therapy as the EHT‐based therapy has displayed impressively improved engraftment and infarct healing, without increasing the incidence of arrhythmias after implantation when compared with transplantation of the hPSC‐CMs alone.^47^, ^49^, ^50^

#### 3.3.3 *Survival and retention*

Low cell survival and long‐term retention are major obstacles hampering cell therapy, especially at the acute phase of MI, which represents one of the most important therapeutic windows to reduce acute cell death and activate the endogenous repair mechanisms.^2^, ^7^, ^23^, ^25^, ^28^, ^51^ To solve this problem, several strategies targeting the following aspects have been developed or under examination: (a) pretreating/engineering the cells before the transplantation to enhance their resistance to stress (eg, treating cells with hypoxic preconditioning and the prosurvival cocktail to induce endogenous cellular survival mechanisms and inhibits major cell death pathways) or genetic engineering of cell‐cycle genes to enhance proliferation of hPSC‐CMs^2^, ^7^, ^22^, ^52^; (b) codelivery of supportive biomaterials (such as biodegradable microparticles^53^) and/or cells (hPSC‐derived ECs and SMCs,^15^, ^54^, ^55^ epicardial cells,^56^ MSCs^57^), or CFs,^16^, ^17^, ^58^ as well as the EHT (cell‐loaded patches and cardiac tissues) to enhance the graft survival, retention, and vascularization.^2^, ^47^, ^49^, ^50^, ^57^ Similar obstacles exist for cell‐free strategies. To retain delivered EVs or secreted factors in the damaged hearts, new solutions are under development, such as combining with biomaterials and making cell mimicking microparticles to target the damaged myocardial tissue^59^; (c) control of cell rejection by various approaches that will be detailed discussed in the “Immune Rejection” section below; and (d) optimizing the host tissue to receive the graft by manipulating the local immune environment of infarcted hearts. Inflammatory responses in acute MI and chronically failing hearts are known to aggravate the infarct injury and impair the survival and retention of transplanted cells. Thus, Li and colleagues^60^ developed an anti‐IL‐1β antibody‐platelet conjugate with the infarcted heart‐homing ability and used it as an anti‐inflammatory agent to treat acute MI. Interestingly, some recent studies have shown that the inflammatory responses also promote infarct healing, including those modulated by the transplanted cells through secreted cytokines.^7^, ^23^, ^61^ In the near future, combining the hPSC‐CM transplantation with the cardiac‐specific immunomodulation approaches need to be tested in both small and larger animal I/R models.

#### 3.3.4 *Arrhythmogenicity*

Grafted hPSC‐CM‐derived ventricular arrhythmias^21^, ^22^, ^62^ is one of the most challenging barriers for their clinical applicability. The mechanism of graft‐induced arrhythmias is unclear. It may arise from the heterogeneous nature of transplanted cells in which a small number of atrial and/or node cells still exist, although current differentiation protocols predominantly generate ventricular like hPSC‐CMs.^22^, ^63^ In addition, considering the implanted hPSC‐CMs underwent maturation in the environment of adult hearts^22^, ^48^ and hPSC‐CM‐derived ventricular arrhythmias reached a peak several days after injection, declined 20 days later,^22^, ^62^ hPSC‐CMs with different maturity may have various degree of functional coupling, ectopic activation, and/or regional conduction, causing the generation of abnormal impulse.^22^, ^45^, ^62^, ^64^ Therefore, it will be critical to test the feasibilities of multiple anti‐arrhythmia approaches in future large animal studies, including identifying proper cell surface markers to prepare highly‐pure ventricular hPSC‐CMs with appropriate maturity for transplantation, fine‐tune of the transplanted cell number, as well as an optimal combination with anti‐arrhythmic drugs.

#### 3.3.5 *Immune rejection*

Due to the allogeneic nature, host immune rejection is the main contributor to the poor cell survival and engraftment of transplanted cells. Immune rejection of hPSC‐derived cardiac lineage cells mainly occurs through major histocompatibility complex (MHC) (or human leukocyte antigen [HLA] in human) system‐mediated adaptive immune system, including T cells and B cells.^25^, ^62^, ^65^ Recent evidence suggests an involvement of the innate immune system in transplantation‐associated immune response, evidenced by the phagocytosis of grafted cells by macrophages via the CD47‐signal‐regulatory protein alpha (SIRPa) pathway.^66^ The immune rejection of transplanted hPSC‐cardiac lineage cells could be partially blunted by immunosuppressive drugs in large animals that underwent cell therapy,^21^, ^22^, ^25^, ^54^ while the side‐effects were detected.^25^ Therefore, new approaches have been and are continuing to be explored in various laboratories. For example, as the immune rejection of foreign cells could be significantly reduced by fine matching of the donor and recipient cells in MHC class I,^65^ several strategies are under developing, including biobanking hPSC lines with a wide diversity of HLA,^6^ creating universal, immune‐compatible donor hPSC lines by overexpressing HLA‐E, and generating humanized organs in other species (eg, pig) that serve as an acceptable donor for patients.^7^, ^49^ More recently, an antibody‐based preconditioning protocol for MHC‐mismatched cell engraftment has been developed, without the need for pharmacological immune suppression.^67^ In sum, remarkable signs of progress have been made to reduce graft immune rejection, and this will continuously act as an important topic to explore so after.

## 4 hiPSC‐CM‐BASED DISEASE MODELING AND DRUG SCREENING

### 4.1 Recent progress

Animal models, mainly transgenic mouse models, do not faithfully reflect the human cardiac pathophysiology as considerable discrepancies exist between species, such as heart anatomy, beat rate, ion channel types, and distribution, etc. Since the pioneering study of LEOPARD syndrome in 2010,^68^ tremendous efforts have been dedicated to the in vitro modeling of heart disease using cardiomyocytes differentiated from patient‐specific hiPSC (hiPSC‐CMs), primarily hereditary channelopathies and cardiomyopathies.^69^

#### 4.1.1 *Cardiac channelopathies*

Cardiac channelopathies are a group of clinical syndromes caused by mutations in genes encoding for cardiac ion channels, including potassium (K^+^), sodium (Na^+^), calcium (Ca^2+^) channels, etc.^70^ A significant number of channelopathies, such as inherited long QT syndrome (LQTS) and catecholaminergic polymorphic ventricular tachycardia (CPVT) have been successfully modeled using patient‐specific hiPSC‐CMs. For example, LQTS3 is characterized by QT‐interval prolongation resulting from a gain‐of‐function mutation in *SCN5A* encoding the Na^+^ channel Nav1.5. hiPSC‐CMs from LQT3 patients replicated the disease phenotypes, such as prolonged action potential duration and aberrant behaviors of Na^+^ channel gating, and the effects can be ameliorated by a Na^+^ channel blocker mexiletine that is an anti‐arrhythmic drug in clinical.^71^, ^72^ In addition, by generating hiPSC‐CMs from patients carrying mutations in *RYR2* gene, which encodes the cardiac ryanodine receptor, a recent study successfully recapitulated the disease phenotypes of CPVT in vitro, illuminated a calmodulin‐dependent protein kinase II (CaMKII)‐dependent pathogenic mechanism of this disease, and identified a highly potent CaMKII inhibitor, myristoylated autocamtide‐2‐related inhibitory peptide, in rescuing the diseased phenotypes.^58^

#### 4.1.2 *Cardiomyopathies*

Cardiomyopathies are a group of disorders linked to impaired structure and functions of heart muscle resulting in HF or sudden cardiac death and are often caused by inherited mutations in genes. Dilated cardiomyopathy (DCM) is a good example of such disorder with a weakened and enlarged heart and has been modeled using hiPSC‐CMs derived from patients with a gene mutation in *TNNT2* that encodes sarcomeric protein cardiac troponin T.^73^ These patient hiPSC‐CMs exhibited reduced contractility, abnormal sarcomeric organization, aberrant Ca^2+^ flux, and increased susceptibility to stress. Furthermore, when treating with the β‐adrenergic blocker metoprolol, identified by the pharmaceutical screen of clinical drugs using this cell model, the diseased phenotypes of DCM hiPSC‐CMs were rescued in culture.^73^ In addition, a recent study has modeled another frequently observed DCM caused by the mutation of the *LMNA* gene that encodes the lamin A/C proteins using hiPSC‐CMs.^74^ The mutant hiPSC‐CMs displayed aberrant calcium homeostasis that led to arrhythmias at the single‐cell level, underlying the abnormal physiological activities of the hearts in patients. Importantly, the arrhythmic phenotypes could be ameliorated by the pharmacological inhibition of the PDGF signaling pathway using several FDA‐approved PDGFRB inhibitors, illuminating a potential novel therapeutic strategy.

### 4.2 Challenges in the field

#### 4.2.1 *Immaturity*

Cardiomyopathy occurs predominantly in the adult stages, and pharmacological study usually requires cardiomyocytes with advanced mature characteristics to faithfully reflect drug response of the adult heart. Thus, the immaturity of hPSC‐CMs mentioned above not only hampers their application in cardiac cell therapy but also emerges as a major obstacle for their application in mincing the true disease phenotype and validate the efficacy of drugs discovered.

#### 4.2.2 *Lack of organized three‐dimensional (3D) structure and microenvironments*

While many researchers have been utilizing monolayer cultured hiPSC‐CMs as 2D models for decades, these systems suffer from a lack of suitable environmental factors including the physiological and anatomical 3D structure of the native heart, active cell‐cell interactions, and crosstalk between the cells and extracellular matrix.^46^ Therefore, it has been reported that hiPSC‐CMs derived from a Barth syndrome patient could only display the disease phenotype in a 3D tissue‐like format but not in 2D culture in peri dishes.^75^

#### 4.2.3 *Lack of proper genetic control*

To precisely define the disease phenotype, researchers need to compare the patient‐derived hiPSC‐CMs with the control cells derived from healthy donors. However, genetic heterogeneity among donors may strongly affect their conclusions, because the difference in phenotypes may be an artifact that merely comes from the diversiform genetic background of the donors, remain a challenge for disease modeling using hiPSC‐CMs.^76^

### 4.3 Toward solutions

#### 4.3.1 *Tissue engineering*

To further enhance the function maturity of hiPSC‐CMs, and to mimic the physiological and anatomical structure of the native heart, it has been well recognized in the field that greater emphasis should be placed on the engineering of 3D myocardial tissues.^58^, ^77^ Cardiac tissue engineering may not only deliver a means to promote cardiomyocyte maturation, but also provide the opportunity to measure contractile function, investigate the effects of mechanical and electrical stimulation in various pathological context, and illuminate the cell‐autonomous or nonautonomous mechanisms that drive the development of certain diseases at a tissue level. An important step to advance the current heart tissue engineering strategy is to combine multiple cutting‐edge techniques, including 3D bioprinting, biochemical stimulation, mechanical stretching, and microfluidic systems.^78^ In addition, it has been shown that an appropriate combination of other cell types, for example, hiPSC‐derived fibroblasts^58^ facilitate EHT construction, enabling the investigation of the molecular and cellular mechanisms underlying exercise‐induced CVPT and drug discovery at a tissue level. Thus, the most appropriate combination of cells and biomaterial for supporting cardiac tissue engineering is of great value and still needs to be defined.

#### 4.3.2 *Genome editing*

With the rapid advances in genome editing technologies, for example, transcription activator‐like effector nucleases (TALENs) and clustered regularly interspaced short palindromic repeats (CRISPR)/CRISPR‐associated (Cas) systems that enable sequence‐specific modification of desired genomic sites in mammalian cells, now researchers can introduce precise mutations into hiPSC lines to create isogenic control cells. They share the same genetic background with the patient‐derived hiPSC‐CMs and only harbor disease‐associated mutations/variants, allowing for stringent analysis of their function.^79^ This will also be instrumental for generating libraries of disease‐specific cardiomyocytes for drug testing and other applications and will continue to be widely used to create cell models of diseases.

#### 4.3.3 *High‐throughput evaluation*

Toward these ultimate goals of understanding and treating heart diseases, the high‐throughput screening will continue to serve as a powerful strategy to discover novel drug chemicals.^80^ The rapid progress of tissue engineering technologies has contributed to the emergence of organ‐on‐a‐chip models for emulating human cardiac diseases. Here, a formation of a reproducible, micro‐sized tissue suitable for high‐throughput manipulation and evaluation is of particular importance.

## 5 CONCLUSION

**FIGURE 1:**
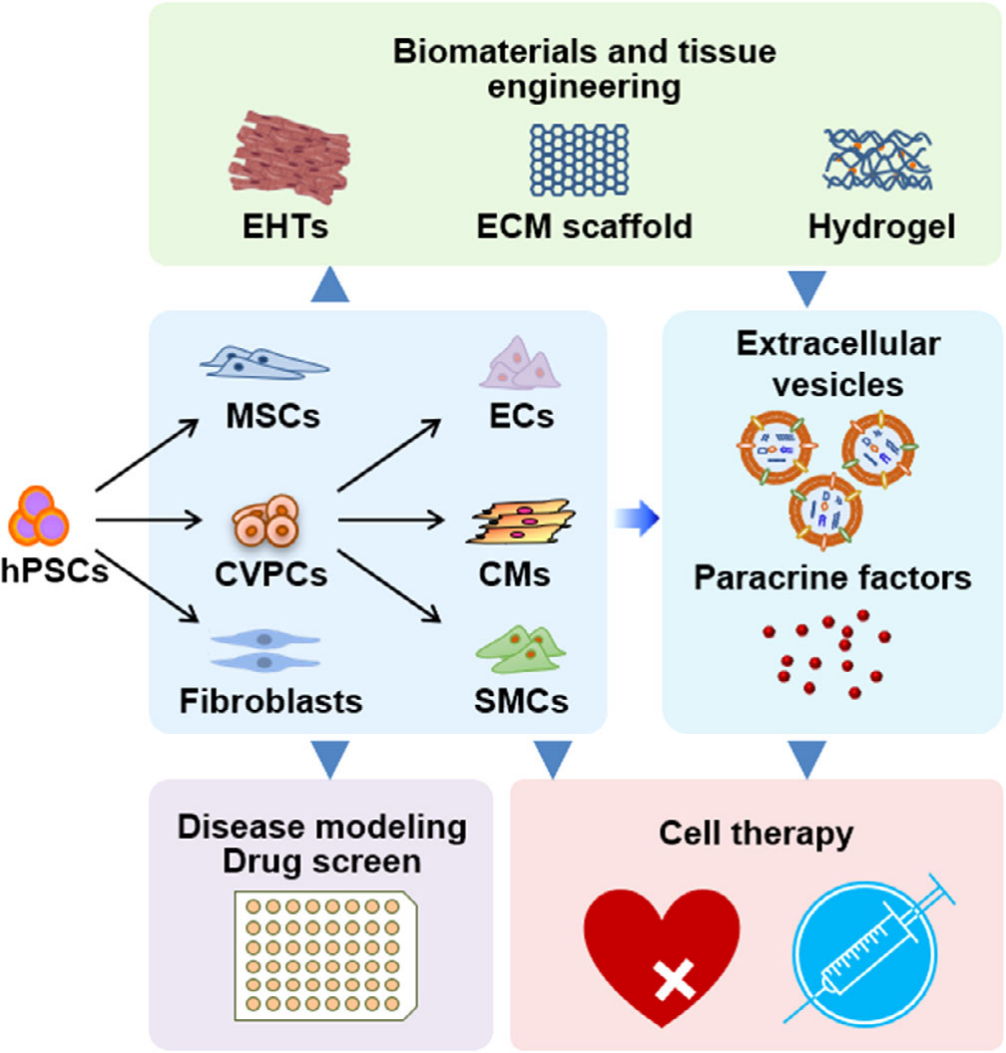
Application of hPSC‐derived cardiac cells in heart disease modeling and repair. Various types of cells, including cardiovascular progenitor cells (CVPCs), cardiomyocytes (CMs), endothelial cells (ECs), smooth muscle cells (SMCs), mesenchymal stem cells (MSCs), and fibroblasts, can be produced from human pluripotent stem cells (hPSCs) in vitro. The resultant hPSC‐derived cells can be subjected to comprehensive heart disease modeling and drug screen applications, or cardiac cell therapy when delivery into the injured heart. Combining an engineered heart tissue (EHT) technique and synthetic or natural extracellular matrix (ECM) scaffolds can assist the engraftment of transplanted cardiac cells. Extracellular vesicles and paracrine factors secreted from hPSC‐derived cardiovascular and supportive cells also benefit heart regeneration by stimulating endogenous repair mechanisms

Substantial progresses have been made toward using hPSC‐CMs not only in the anticipated application of cell‐replacement and cell‐free therapy but also in reproducing the pathological phenotypes of many cardiac diseases of genetic forms or caused by abnormal physiological insults (Figure 1). However, considering the complicated nature of the development and maturation of hPSC‐CM function, and incomplete knowledge of the underlying molecular mechanisms that we understand, the concerted action of both electrophysical and mechanical apparatuses presents within the cell remain to be more thoroughly investigated. Moreover, mechanisms of cell‐host interactions, the crosstalk between individual transplanted cells, and the cardiac regeneration need to be comprehensively dissected. These mechanistic insights will be critical for the development of new strategies to overcome the obstacles in cell‐based cardiac repair, which is of great importance for moving the cell therapy and cell‐free therapy toward clinical transplantation. In combination with either genetic loss‐of‐function approaches to find therapeutic targets, or small molecule libraries to find novel lead compounds for drug discovery, this humanized cell‐based strategy may represent a powerful platform, in conjunction with animal disease models and the rapid progressing techniques in cardiac tissue engineering, to advance the understanding and treatment of heart diseases.

## CONFLICT OF INTEREST

The authors declared no potential conflicts of interest.

## AUTHOR CONTRIBUTIONS

Q.L., J.W., Q.W.: manuscript writing; H.T.Y., N.C.: conception and design, manuscript writing, financial support, and final approval of the manuscript.

6

## Data Availability

Data sharing is not applicable to this article as no new data were created or analyzed in this study.
